# Integrating germline and somatic variation information using genomic data for the discovery of biomarkers in prostate cancer

**DOI:** 10.1186/s12885-019-5440-8

**Published:** 2019-03-14

**Authors:** Tarun Karthik Kumar Mamidi, Jiande Wu, Chindo Hicks

**Affiliations:** Department of Genetics and the Bioinformatics and Genomics Program, Louisiana State University Health Sciences Center, School of Medicine, 533 Bolivar, New Orleans, LA 70112 USA

**Keywords:** Germline somatic mutations genomic analysis prostate Cancer

## Abstract

**Background:**

Prostate cancer (PCa) is the most common diagnosed malignancy and the second leading cause of cancer-related deaths among men in the United States. High-throughput genotyping has enabled discovery of germline genetic susceptibility variants (herein referred to as germline mutations) associated with an increased risk of developing PCa. However, germline mutation information has not been leveraged and integrated with information on acquired somatic mutations to link genetic susceptibility to tumorigenesis. The objective of this exploratory study was to address this knowledge gap.

**Methods:**

Germline mutations and associated gene information were derived from genome-wide association studies (GWAS) reports. Somatic mutation and gene expression data were derived from 495 tumors and 52 normal control samples obtained from The Cancer Genome Atlas (TCGA). We integrated germline and somatic mutation information using gene expression data. We performed enrichment analysis to discover molecular networks and biological pathways enriched for germline and somatic mutations.

**Results:**

We discovered a signature of 124 genes containing both germline and somatic mutations. Enrichment analysis revealed molecular networks and biological pathways enriched for germline and somatic mutations, including, the PDGF, P53, MYC, IGF-1, PTEN and Androgen receptor signaling pathways.

**Conclusion:**

Integrative genomic analysis links genetic susceptibility to tumorigenesis in PCa and establishes putative functional bridges between the germline and somatic variation, and the biological pathways they control.

**Electronic supplementary material:**

The online version of this article (10.1186/s12885-019-5440-8) contains supplementary material, which is available to authorized users.

## Background

Prostate cancer (PCa) is the most common solid tumor and the second most common cause of cancer-related death in men in the United States [1]. In 2017, there were an estimated 180,890 new cases of PCa and 26,120 men died of the disease [1]. The most well supported risk factors include age, ethnicity, family history and genetics. Progression from genetic susceptibility to tumorigenesis involves both the germline and the somatic variation [2]. However, elucidating the possible oncogenic interactions between germline and somatic mutations in tumorigenesis remains elusive. Exploring the two genomes jointly could lead to a better understanding of how cancer risk alleles contribute to carcinogenesis.

Advances in high-throughput genotyping and reduction in genotyping costs have enabled discovery of genetic susceptibility variants (herein called germline mutations) associated with an increased risk of developing PCa using genome-wide association studies (GWAS) [3]. We recently published a comprehensive catalogue of genetic susceptibility variants primarily single nucleotide polymorphisms (SNPs) and genes associated with an increased risk of developing PCa from GWAS [3]. These genetic variants are providing valuable clues about the genetic susceptibility landscape of PCa. To infer the causal association between gene expression and the disease and to establish putative functional bridges between GWAS discoveries and biological pathways, we integrated GWAS information with gene expression data [4, 5]. However, information linking genetic variation with acquired somatic variation the main driver of tumorigenesis is lacking. This knowledge gap has impeded translation of GWAS discoveries into clinical practice to guide treatment decisions.

The recent surge of next generation sequencing of tumor genomes has enabled discovery of recurrent somatic mutations and led to expanded molecular classification of PCa [6]. Large multicenter efforts such as The Cancer Genome Atlas (TCGA) and the International Cancer Genome Consortium (ICGC) have performed a series of detailed analyses of somatic mutations and other genomic alterations driving tumorigenesis [6, 7]. However, to date, the information on acquired somatic mutations has not been leveraged and integrated with GWAS information to establish the possible link between genetic susceptibility and tumorigenesis. Germline mutations such as SNPs can function as oncogenic modifiers or co-oncogenes, thus, could determine what complementary somatic mutations are required for full malignant transformation. Therefore, integrating germline with somatic mutation information has the promise of identifying genes, molecular networks and biological pathways driving PCa development and progression. Such markers and pathways could be used for the development of novel targeted therapies and novel early intervention strategies critical to the realization of both precision medicine and precision prevention.

The objectives of this study were: 1) to investigate the potential link between genetic susceptibility from GWAS and tumorigenesis from sequencing in the TCGA and 2) to discover and characterize the molecular networks and biological pathways enriched for germline and somatic mutations. Our working hypothesis was that genes containing germline mutations associated with an increased risk of developing PCa also harbor recurrent somatic mutations acquired during tumor formation. We further hypothesized that genes containing germline mutations are functionally related with genes containing acquired somatic mutations and interact in molecular networks and biological pathways driving tumorigenesis. We addressed these hypotheses by integrating information on germline mutations and genes associated with and increased risk of developing PCa derived from GWAS with information on acquired somatic mutations derived from next generation sequencing of tumor genomes in TCGA, using transcriptome data from the TCGA as the intermediate phenotype. We performed enrichment analysis to identify molecular networks and biological pathways enriched for germline and somatic mutations. This novel integrative genomics approach was designed to help determine whether and to what extent pathways involved in cancer risk may also be involved in cancer development and progression. For clarity, we have considered SNPs associated with an increased risk of developing PCa discovered using GWAS as germline mutations. Somatic mutations are acquired genetic alterations during tumor formation discovered by sequencing the tumor samples. Throughout this report we have used the gene as the unit of association and its expression data as the intermediate phenotype linking germline with somatic variation.

## Methods

### Germline mutations and associated genes

Advances in high-throughput genotyping and reduction in genotyping costs have enabled discovery of germline mutations and genes associated with an increased risk of developing PCa using GWAS [3]. We have previously developed and published a comprehensive catalogue of germline mutations and genes from GWAS and integrated GWAS information with gene expression data to infer the causal association between gene expression and PCa [4, 5]. Building on this line of research, in this study we used germline mutations and associated genes from the catalogue we developed using publicly available data obtained from published reports on GWAS and the websites hosting supplementary data for the respective reports [3–5]. The methods of GWAS data collection have been reported in our previous reports [3–5] and were based on the guidelines proposed by the Human Genome Epidemiology Network for systematic review of genetic associations which is the standard [8–12]. The authenticity of the germline mutations and gene names were further verified using the Single Nucleotide Polymorphism Database (dbSNP), a free public archive for genetic variation within and across different species developed and hosted by the National Center for Biotechnology Information in collaboration with the National Human Genome Research Institute [13] and the Human Genome Gene Nomenclature Committee (HGNC) database [14]. A complete list of germline mutations and the genes they map to including original reports from which the information was derived is presented in Additional file 1: Table S1 provided as supplementary material.

### Somatic mutations information

TCGA has used next generation sequencing technology to sequence the cancer genomes and has provided detailed analysis of somatic mutations [6]. All the samples in the TCGA were processed using the same techniques and technology platform to eliminate bias. We downloaded information on somatic mutations on 495 PCa patients from the TCGA via the Genomics Data Commons https://gdc.cancer.gov/. The information included 12,876 somatically altered genes and 31,596 somatic mutations. We computed the somatic mutation frequency in samples to determine the distribution of mutations and to identify the genes which are highly mutated. This was done to assess the heterogeneity in the mutational processes in cancer. From these analysis, we created a comprehensive list of mutated genes across samples. A complete list of somatically mutated genes along with somatic mutations is presented in Additional file 2: Table S2 provided as supplementary data to this report.

### Gene expression data

Gene expression data derived from RNA-seq was downloaded from TCGA using Genomics Data Commons (GDC) data transfer tool along with clinical information. A total of 547 patient samples were downloaded. The distribution of the samples was *N* = 495 tumors and *N* = 52 normal samples. All the sample were processed on the same illumine platform to allow for consistence in gene expression levels and eliminate batch effects. The data matrix was filtered to remove rows with missing data, such that each row has at least ≥30% data using cpm (counts per million) filter (> 0.5) in R. The resulting data set was normalized by TMM (The trimmed mean of M-values) normalization method and then transformed by Voom, using Limma package implemented in R [15]. The normalized data contained 18,428 probes and was used in the analysis. The probe IDs and gene symbols and names were matched for interpretation using the Ensemble database, a database used for gene annotation of sequencing experiments and sequencing technology platforms.

#### Data analysis

**Fig. 1:**
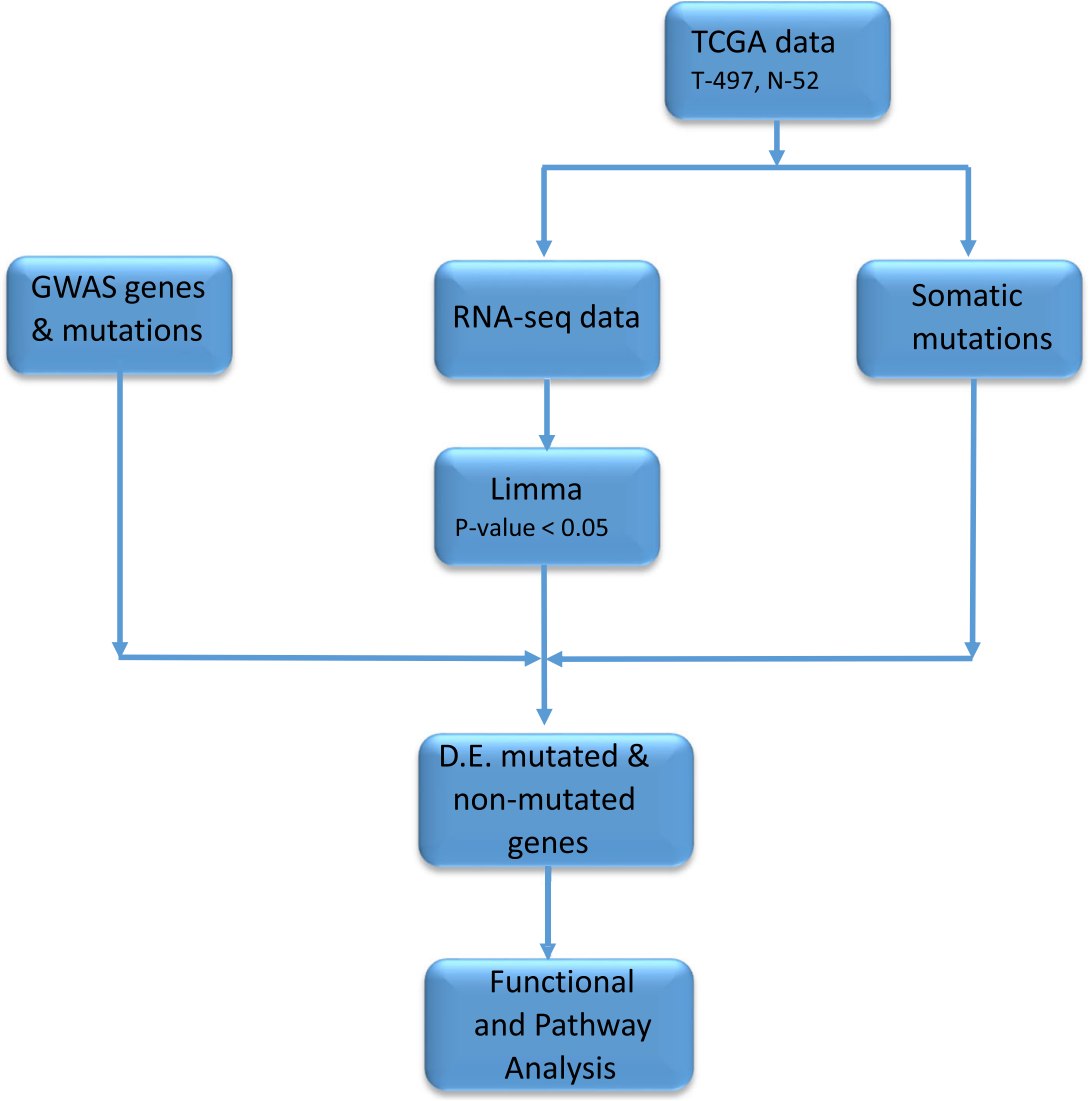
Design and workflow for integrative analysis combining germline with somatic mutation information using gene expression data. RNA-seq read count data and somatic information were downloaded from the cancer genome atlas (TCGA). Germline mutation information was manually curated from GWAS studies. Limma (R) package was used for the discovery of differentially expressed (D.E.) mutated and non-mutated genes. Ingenuity Pathway Analysis (IPA) was used for functional analysis and discovery of molecular networks and biological pathways enriched for germline and somatic mutations

The project design and data analysis workflow are presented in Fig. 1. We performed whole genome analysis comparing gene expression levels between patients diagnosed with tumors and matched control samples using the Limma package implemented in R to identify all significant differentially expressed genes distinguishing tumors from control samples [15]. This unbiased approach was carried out to discover, germline and somatically mutated genes as well as non-mutated genes. We used the false discovery rate (FDR) procedure to correct for multiple hypothesis testing [16]. The genes were ranked on *P*-values and the FDR. We performed enrichment analysis using Ingenuity Pathway Analysis (IPA) software [17]. Using IPA, the most highly significantly differentially expressed genes distinguishing patients with tumors from control samples were mapped onto networks and canonical pathways. The probability scores and the log *P*-values were calculated to assess the likelihood and reliability of correctly assigning the genes to the correct molecular networks and biological pathways. A false discovery rate was used to correct for multiple hypothesis testing in pathway analysis. The predicted molecular networks and biological pathways were ranked based on z-scores and log *p*-values; respectively. Gene ontology (GO) [18] analysis as implemented in IPA, was performed on the sets of differentially expressed genes to characterize the functional relationships among sets of differentially expressed genes. Genes were classified according to the molecular functions, biological processes and cellular components in which they are involved.

## Results

### Differential expression of mutated and non-mutated genes

**Fig. 2:**
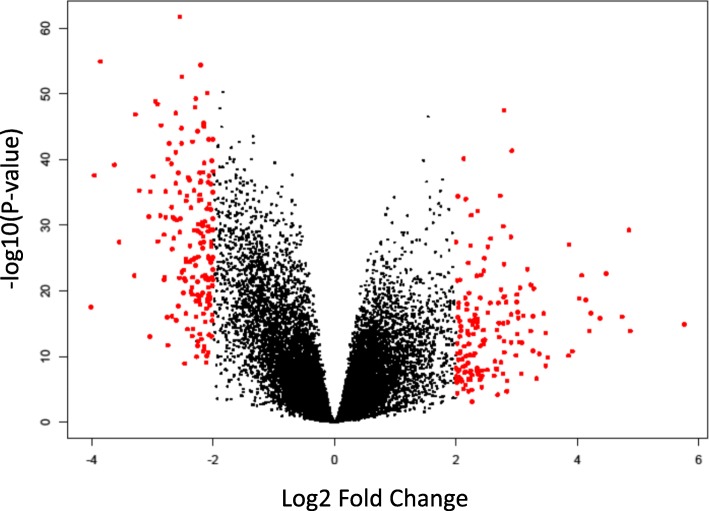
Volcano plot showing results of supervised analysis for the discovery of germline and somatic mutated and non-mutated genes generated by the Limma package in R to find differentially expressed markers (red dots represent the genes with −2 ≥ and ≥ 2 log fold change)

To discover significantly differentially expressed mutated and non-mutated genes distinguishing patients with tumors from matched control samples, we performed whole genome analysis comparing expression levels of the 18,333 genes between tumors and matched control samples. We hypothesized that gene expression levels significantly differ between patients with tumors and control samples. We sought to discover signatures of significantly differentially expressed somatic mutated and non-mutated genes as described in the analysis section. A visual representation of the results of differential expression analysis as determined by the log *p*-value and log2 fold changes are presented in a volcano plot in Fig. 2. Since the filter with log fold change results in fewer number of gene signatures, we used p-value for further analysis.

After controlling for multiple hypothesis testing, whole genome analysis revealed a signature of 6912 significantly (*P* < 0.05) differentially expressed somatic mutated genes, of which 6041 genes were highly significantly (*P* < 0.01) differentially expressed. In addition, whole genome analysis produced a signature of 5609 significantly (P < 0.05) differentially expressed non-mutated genes, of which 4879 genes were highly significantly (P < 0.01) differentially expressed. Among the most highly significantly differentially expressed somatically mutated genes with high mutation frequency were *TP53, SYNE1, FOXA1, LRP1B, FAT3, SPOP, DNAH17, FAT4, MACF1, AHNAK2, ANK2, PTEN, DST, DCHS2, MXRA5, MALAT1, VPS13D, HECTD4, FREM2, MYO15A.* A complete list of significantly differentially expressed somatically mutated genes including the mutation frequencies and the non-mutated genes along with their estimates of *p*-values and false discovery rates are presented in Additional file 3: Table S3 for somatic mutated genes and Additional file 4: Table S4 for non-somatic mutated genes provided as supplementary data to this report.

### Linking germline mutated genes with PCa and somatic information

To determine whether genes containing germline mutations associated with an increased risk of developing PCa derived from GWAS are involved in PCa, we performed two analysis strategies. First, we evaluated the genes containing germline mutations against the set of all genes found to be differentially expressed following whole genome analysis. We hypothesized that genes containing germline mutations are significantly differentially expressed between patients with tumors and control samples. The goal of this analysis was to infer the potential causal association between GWAS information and tumorigenesis using gene expression data as the intermediate phenotype. We sought to discover a signature of germline mutated genes distinguishing tumors from controls.

In the second step, we evaluated the genes containing germline mutations against the set of significantly differentially expressed genes containing somatic mutations. We hypothesized that significantly differentially expressed genes containing germline mutations also harbor acquired somatic mutations. The goal of this analysis was to establish the link between germline and somatic mutation information using genes as the organizing units and gene expression as the intermediate phenotype. We sought to discover a gene signature containing both germline and somatic mutated genes distinguishing tumor samples from control samples.

**Fig. 3:**
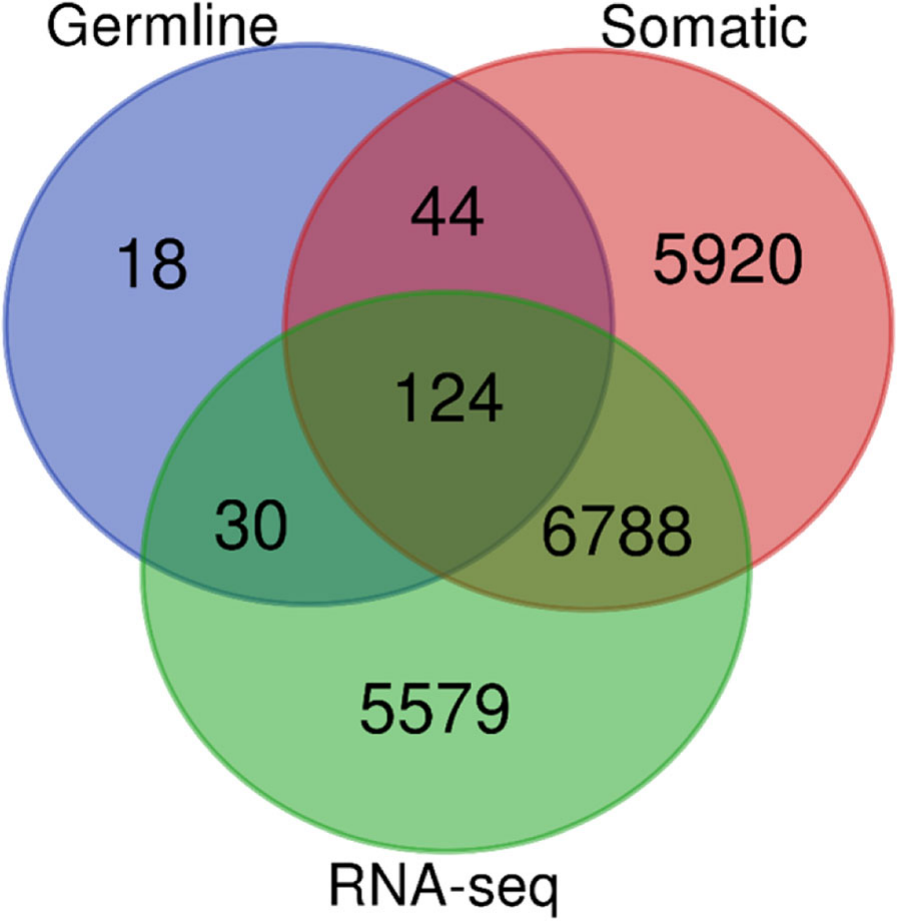
Venn diagram showing differentially and non-differentially expressed germline and somatic mutated and non-mutated genes. Middle intersections shows 124 genes containing both germline and somatic mutations and are also significantly expressed with RNA-seq dataset. *Germline* – indicates genes with germline genetic susceptibility variants, *Somatic* – indicates genes with somatic mutations from TCGA, *RNA-seq* **–** indicates differentially expressed gene signatures from TCGA expression data

The results of these analyses are presented in a venn diagram in Fig. 3. Out of the 304 genes containing germline mutations evaluated, 216 genes matched with gene symbols in RNA-Seq data from TCGA. From this set of genes, a total of 168 genes contained germline and somatic mutations (Fig. 3). Among them, 124 genes were significantly differentially expressed distinguishing tumors from controls (Fig. 3). The remaining 44 genes containing both germline and somatic mutations were not significantly differentially expressed (Fig. 3). In addition, 30 genes containing germline mutations were significantly differentially expressed, but did not contain somatic mutations (Fig. 3). A small number (18) out of genes containing germline mutations did not contain somatic mutations and were not significantly differentially expressed (Fig. 3). The discrepancy between the 304 genes discovered in GWAS reports and the 216 genes matching sequence data can be partially explained by the discrepancies in annotation inherent in GWAS data and Ensemble database. Some of the genetic variants in GWAS are reported to map to nearby genes and not to the actual genes. Here we considered germline mutations and genes as reported in the GWAS reports we reviewed to avoid misrepresentation of the results in original reports. Under such conditions, the observed discrepancies or outcome should be expected.

Evaluation of somatically mutated genes, revealed 6788 genes containing somatic mutations only which were significantly differentially expressed. A total of 5920 genes containing only somatic mutations were found to be not significantly differentially expressed (Fig. 3). Further evaluation of the data revealed 5579 genes without any mutations, but were significantly differential expressed (Fig. 3).

### Distribution of germline and somatic mutations

To assess the frequency distribution of germline and somatic mutations in each gene, we counted the number of both germline and somatic alterations per gene. In GWAS, evidence of strong genome-wide association is determine by *P* ≤ 10^− 8^ and validated by replication of discovered genetic variants in multiple independent studies. However, because PCa is a polygenic disease involving many loci, each with a small effect, but likely acting in concert with each other to affect disease phenotypes, here we evaluated genes containing genetic variants with strong association as well as those with weak to moderate association with PCa as described in the methods section.

The results showing a list of significantly differentially expressed genes containing both germline and somatic mutations are shown in Table 1. (Note that only genes containing the genetic variants strongly associated with PCa and replicated in multiple independent studies are presented in Table 1). Also presented in the table are the genetic variants with GWAS association *p*-values, gene expression p-values, FDR and the frequency distribution of germline and somatic mutations in each gene. The analysis revealed 49 genes containing genetic variants associated with an increased risk of developing PCa, which also contained somatic mutations (Table 1). The number of germline mutations varied considerably ranging from 1 to 15 germline mutations per gene, while the number of somatic mutations ranged from 1 to 29 (Table 1). About 32 genes contained only one germline mutation, whereas only 4 genes contained one somatic mutation, suggesting that somatic mutations occur at a higher frequency than germline mutations (Table 1). The genes *NKX3–1, KLK3, ZNF827, EHBP1, HOXB13, PDLIM5, POU5F1B, FGF2, TBX5, SLC25A37, MYC, RFX6, WDR11, JAZF1, KLK2, SLC45A3* and SLC22A3 had more than one germline mutation (Table 1). Most of these genes have been implicated in PCa [4, 5].

**Table 1:** List Genes containing germline and somatic mutations that were significantly differentially expressed between tumors and controls

Genes	Region	SNP ID	GWAS *P*-value	EXP *P*-Value	FDR	Germline mutations	Somatic mutations
*LRP1B*	2q21.2	rs10210358	2.00E-06	5.57E-26	2.30E-24	1	29
*PKHD1*	6p21.2	rs10498792	3.00E-06	0.004895	0.008362	1	11
*TMPRSS2*	21q22.3	rs1041449	3.00E-08	7.18E-06	1.92E-05	1	9
*DNAH5*	5p15.2	rs4463179	2.00E-06	5.10E-18	7.01E-17	1	8
*MYO6*	6q14.1	rs9443189	4.00E-08	1.83E-08	7.13E-08	1	7
*ATF7IP*	12p13.1	rs3213764	2.00E-09	9.80E-06	2.56E-05	1	7
*TBX3*	12q24.21	rs11067228	1.00E-14	0.002485	0.004448	1	7
*NKX3–1*	8p21.2	rs13272392	4.00E-34	2.12E-22	5.29E-21	4	6
*KLK3*	19q13.41	rs2739472	9.00E-186	1.12E-17	1.48E-16	15	6
*BCL11A*	2p16.1	rs2556375	6.00E-19	8.61E-16	8.83E-15	1	6
*GLI2*	2q14	rs11122834	5.00E-06	2.11E-10	1.06E-09	1	6
*ZNF827*	4q31.22	rs56935123	4.00E-09	9.84E-05	0.00022	2	6
*EHBP1*	2p15	rs2430386	9.00E-12	0.016795	0.026074	6	6
*HOXB13*	8q24.21	rs188140481	6.00E-34	1.57E-28	9.23E-27	2	5
*PPFIBP2*	11p15.4	rs12791447	4.00E-08	2.55E-11	1.46E-10	1	5
*NOTCH4*	6p21.3	rs3096702	4.78E-09	1.70E-06	4.96E-06	1	5
*IL16*	15q26.3	rs7175701	9.8E-08	0.005231	0.008879	1	5
*DDHD1*	14q22.1	rs8008270	2.00E-14	0.023066	0.03488	1	5
*PDLIM5*	4q22	rs17021918	4.2E-15	2.67E-23	7.52E-22	2	4
*POU5F1B*	8q24.21	rs16901979	1.00E-16	4.98E-19	7.86E-18	2	4
*EBF2*	8p21.2	rs11135910	8.00E-11	9.81E-10	4.50E-09	1	4
*TLR4*	9q33.1	rs6478343	2.00E-08	7.99E-05	0.000181	1	4
*TNS3*	7p12.3	rs56232506	9.00E-09	0.009192	0.014986	1	4
*FGFR2*	10q26.12	rs10886902	2.00E-53	3.68E-25	1.36E-23	2	3
*NLGN3*	Xq13.1	rs4844289	1.00E-09	1.44E-24	4.91E-23	1	3
*MLPH*	2q37.2	rs2292884	4.00E-08	1.06E-20	2.11E-19	1	3
*FAM111A*	11q12.1	rs1938781	1.10E-10	1.75E-19	2.94E-18	1	3
*RAD51B*	14q23	rs7141529	2.77E-10	3.57E-12	2.28E-11	1	3
*ZNF652*	17q21.32	rs7210100	3.4E-13	2.05E-11	1.18E-10	1	3
*ADAM15*	1q21.3	rs1218582	2.00E-08	2.34E-11	1.34E-10	1	3
*TBX5*	12q24.1	rs1270884	6.75E-11	8.52E-07	2.61E-06	2	3
*CNNM2*	10q24.32	rs3850699	5.00E-10	0.001153	0.002181	1	3
*FERMT2*	14q22.1	rs8008270	1.78E-14	1.98E-26	8.69E-25	1	2
*SLC25A37*	8p21.2	rs4614003	1.00E-15	2.31E-17	2.93E-16	2	2
*FOXP4*	6p21.1	rs1983891	7.6E-08	2.60E-13	1.93E-12	1	2
*NGFR*	17q21.32	rs11650494	2.00E-09	6.87E-12	4.23E-11	1	2
*FAM111B*	11q12.1	rs1938781	1.10E-10	1.08E-11	6.47E-11	1	2
*MYC*	8q24.21	rs10505477	7.00E-21	7.25E-10	3.39E-09	8	2
*NAALADL2*	3q26.31	rs78943174	4.00E-08	9.47E-08	3.34E-07	1	2
*KCNN3*	1q21.3	rs1218582	1.95E-08	9.70E-08	3.42E-07	1	2
*MDM4*	1q32	rs4245739	2.01E-11	0.003357	0.005885	1	2
*SERPINA3*	14q32.13	rs8023057	2.00E-15	0.010238	0.016542	1	2
*RFX6*	6q22.31	rs339331	2.00E-12	0.015259	0.02389	2	2
*SHROOM2*	Xp22.2	rs2405942	2.37E-10	0.017786	0.027489	1	2
*WDR11*	10q26.13	rs10749415	9.00E-25	0.032303	0.047546	2	2
*JAZF1*	7p15.2	rs1080784	2.96E-10	9.20E-30	6.55E-28	7	1
*KLK2*	19q13.33	rs1354774	6.00E-20	1.78E-23	5.15E-22	5	1
*SLC45A3*	1q32.1	rs12409639	2.36E-19	1.12E-11	6.71E-11	4	1
*SLC22A3*	6q25.3	rs4646284	3.2E-52	0.000156	0.000339	4	1

Interestingly, genes containing germline mutations with moderate to weak associations were also found to be somatically mutated, some of which were found to be highly somatically mutated. A complete list of germline and somatically mutated genes found to be associated with PCa in this report is presented in Additional file 5: Table S5 provided as supplementary data to this report.

### Enrichment analysis of molecular networks and biological pathways

To gain insights about the broader biological context in which germline and somatically mutated genes operate, we performed network and pathways analysis. We hypothesized that genes containing germline and somatic mutations are functionally related and interact with one another in molecular networks and biological pathways. We sought to identify molecular networks and biological pathways enriched for germline and somatic mutations. To ensure reliability of the networks, we kept only the genes connected with solid lines and have at least two or more connections.

**Fig. 4:**
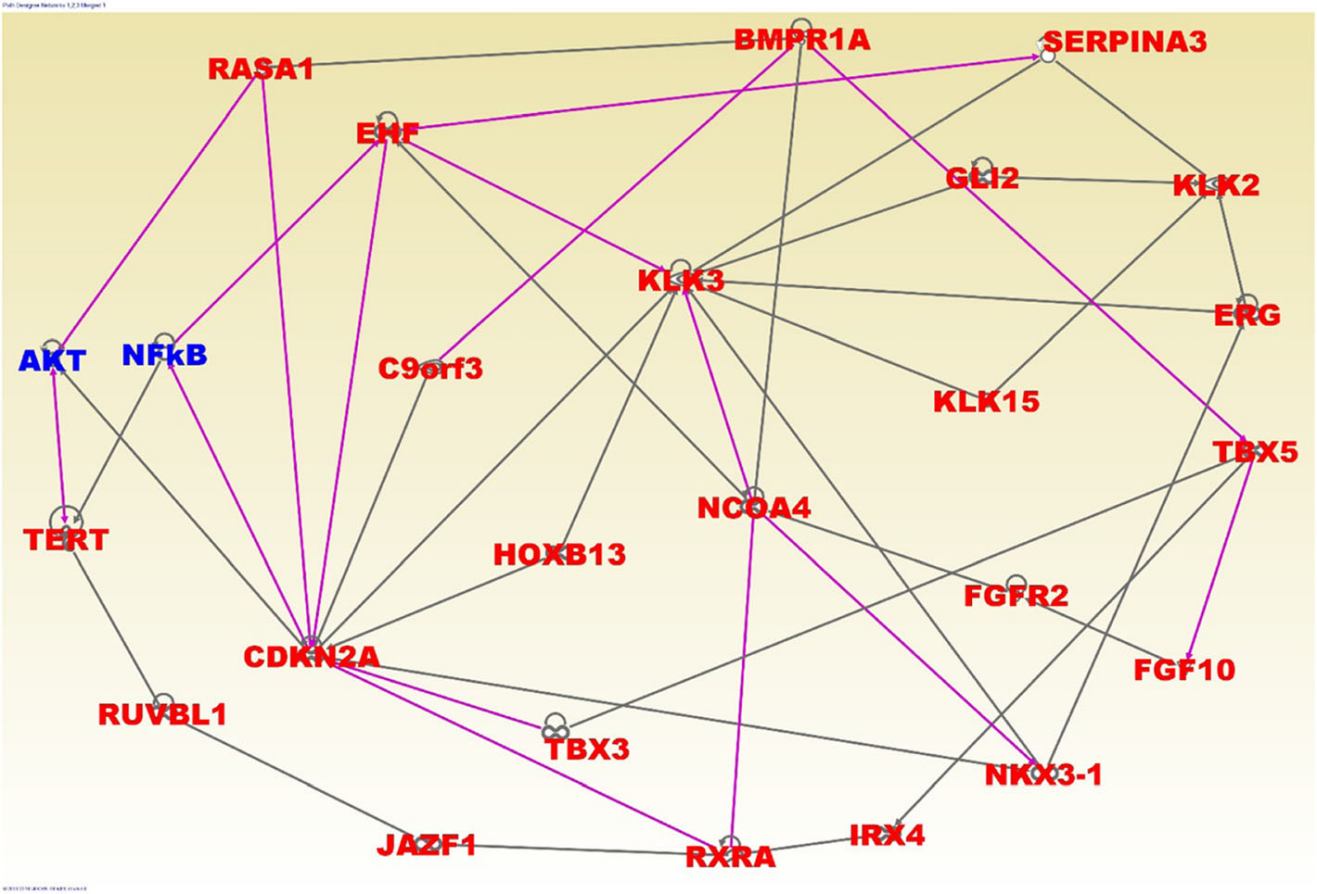
Molecular networks showing interactions among genes containing both germline and somatic mutations (in red font) and literature associated (in blue font). The nodes show the gene names and the solids lines show functional relationships

**Fig. 5:**
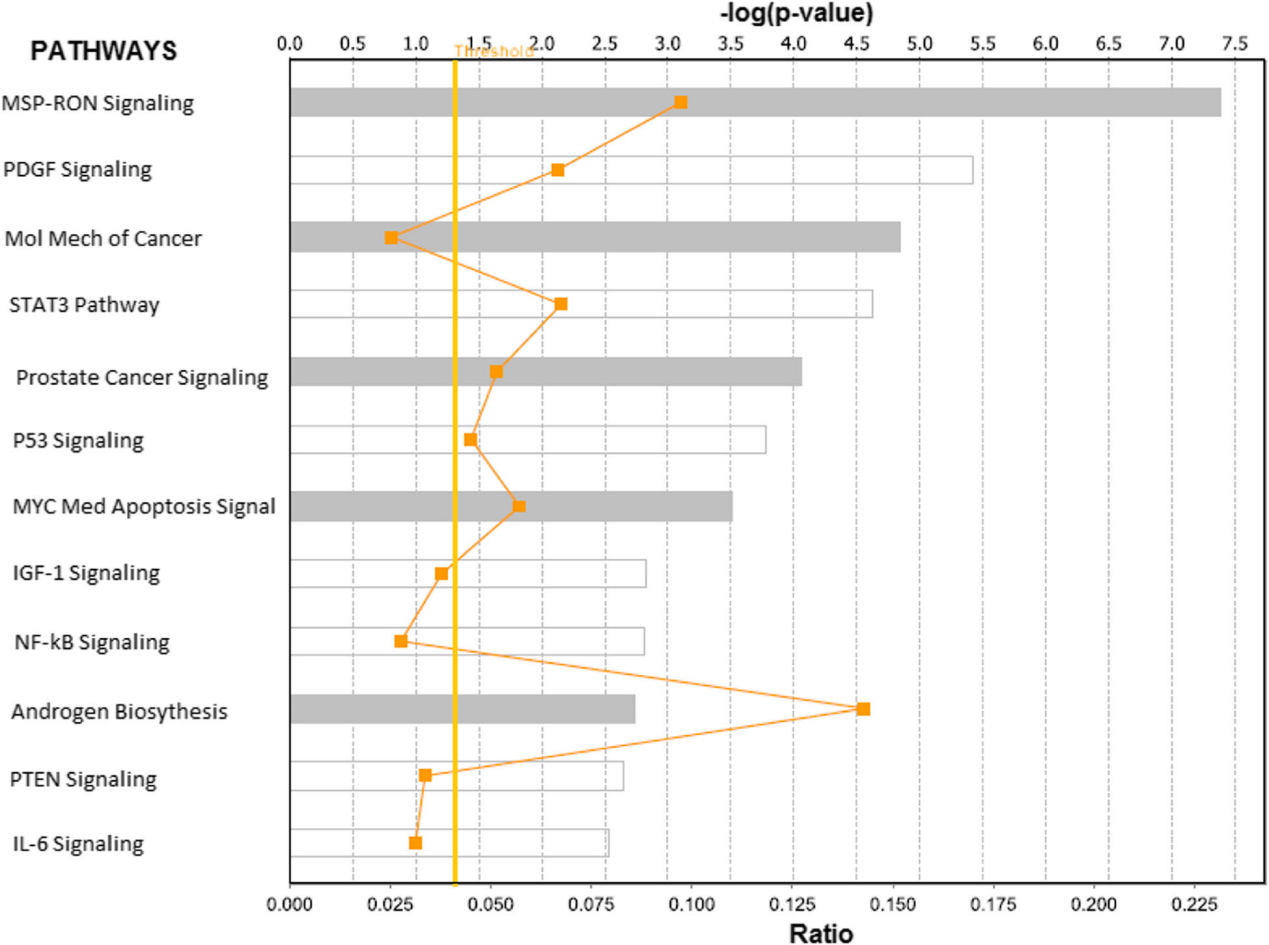
Biological Pathways enriched for germline and somatic mutations. The pathways were constructed using genes containing both germline and somatic mutations. The vertical yellow line indicates the threshold level for declaring significance as determine by the log *p*-value. The zigzagging orange line indicates the ratio of molecules assigned to the pathway from input genes to the original number genes in the pathway

The results of network and pathway analysis are shown in Figs. 4 and 5; respectively, for genes containing both germline and somatic mutations. Network analysis revealed functional relationships and interactions among genes containing germline and somatic mutations (Fig. 4, red fonts). Network analysis of germline and somatic mutated genes revealed genes predicted to be involved in gene expression (*JAZF1, FOXP4, PDLIM5, PHF19, NUCKS1, RxRA, TBX3, TBX5, TERT, RUVBL1, IRX4*), cancer (*C9ORF3, CDKN2A, ERG, HOXB13, KLK2, KLK3, KLK15, MDM4, NKX3–1, SERPINA3, TCF, MLPH, ADCY5, MIRLET7*), cell cycle (*IKZF2, KCTD11, KLF17, POU5F1, PRDM15*), cell death (*ALKBH7, LMTK2, MUC15, NUDT11, PPP1R1C, PTPN6, RAD51B, SLC22A3, SLC35A1, STAT3, TGFBR1,THADA, ZNF300, ZNF652*); and DNA repair replication and repair (*ALDH1L1, CNGB3, DMKN, DNAJB7, NUDT9, PDXP, PP2D1, PPFIBP2, RAB28, SET, SETBP1, SHISA3, THBS4, ZMYM5, ZNF445*).

Interestingly, network analysis revealed interactions among germline and somatically mutated genes containing germline mutations with strong associations to PCa, including, *KLK3, C9orf3, GLI2, KLK15, JAZF1, IRX4, NKX3–1, FGF10, RASAI, TBX5* and *TERT* (Fig. 4). Additionally, network analysis revealed interactions among germline and somatically mutated genes containing germline mutations with weak to moderate association with PCa.

Pathway analysis revealed biological pathways enriched for germline and somatic mutations, predicted to be highly significantly involved in prostate cancer (Fig. 5). Among the top most highly significant pathways (*P* < 1.0 × 10^− 5^) included the pathways involved in MSP-RON, PDGF, molecular mechanisms of cancer, STAT3, prostate cancer, P53, MYC mediated apoptosis, IGF-1, NF-kB, Androgen biosynthesis, PTEN and IL-6 signaling pathways (Fig. 5). Interestingly, both the genes containing genetic variants with strong association were found to be functionally related and interacting with genes containing genetic variants with weak to moderate association.

One of the major concerns and limitations of GWAS is that most of the variants associated with an increased risk of developing PCa identified thus far confer relatively small increments in risk, and explain only a small proportion of the phenotypic variation, leading many to question how the remaining, ‘missing’ variation can be explained [19–22]. Additionally many of the GWAS identified variants may not be causal [21, 22]. Therefore, focusing on only the genes containing germline and somatic mutations, may miss important somatically mutated driver genes and pathways. To address this critical knowledge gap, we performed additional network and pathways enrichment analysis combining the set of germline and somatically mutated genes with highly somatically mutated genes containing no germline mutations. We hypothesized that germline mutated genes are functionally related with highly somatically mutated genes without germline mutations. We further hypothesized that germline mutated genes interact with highly somatically mutated genes containing no germline mutations in molecular networks and biological pathways enriched for both genetic alterations.

**Fig. 6:**
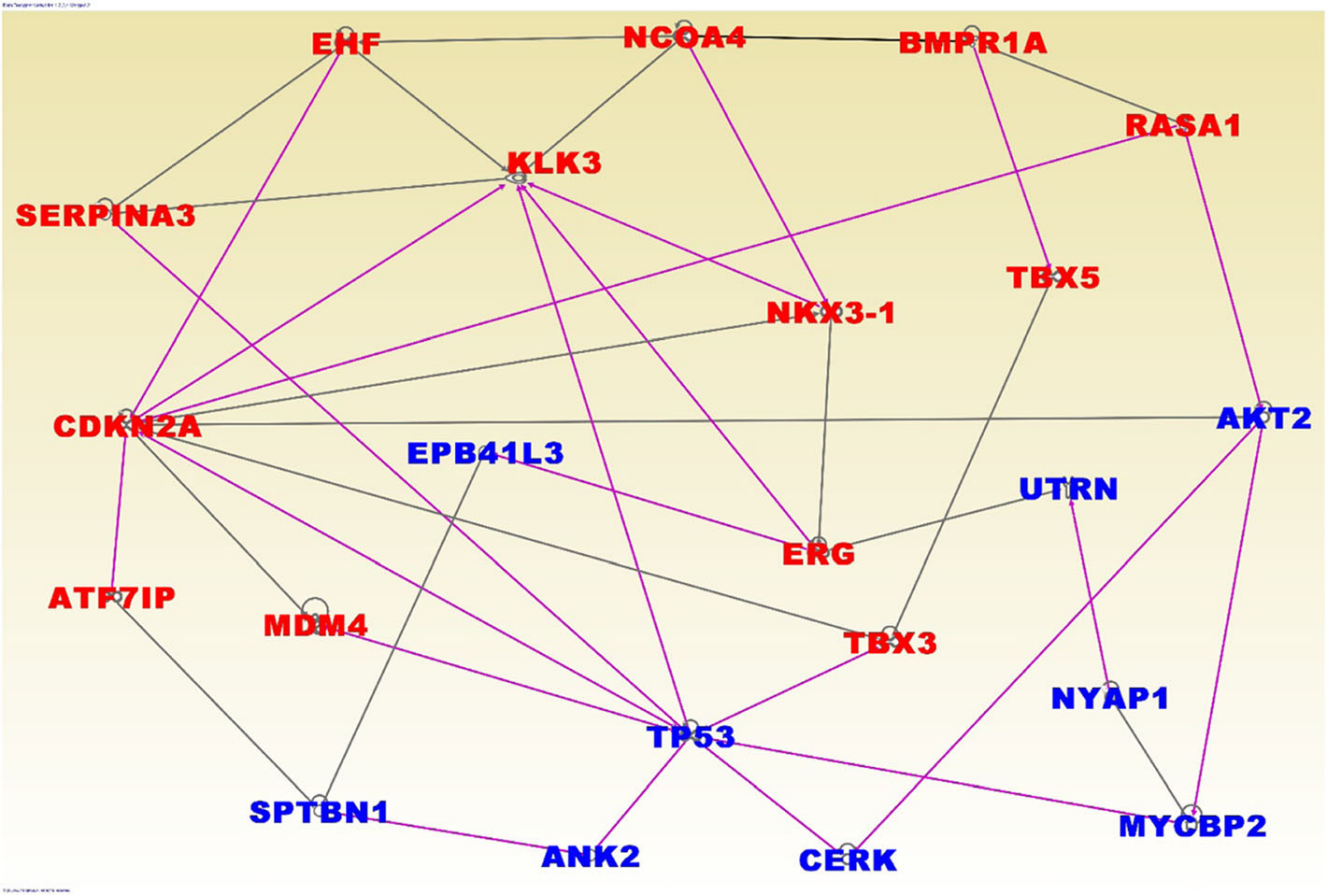
Molecular networks showing interactions among genes containing germline mutations (in red font) and highly somatically mutated genes without germline mutations (in blue font). The nodes show the gene names and the solid lines show functional relationships

**Fig. 7:**
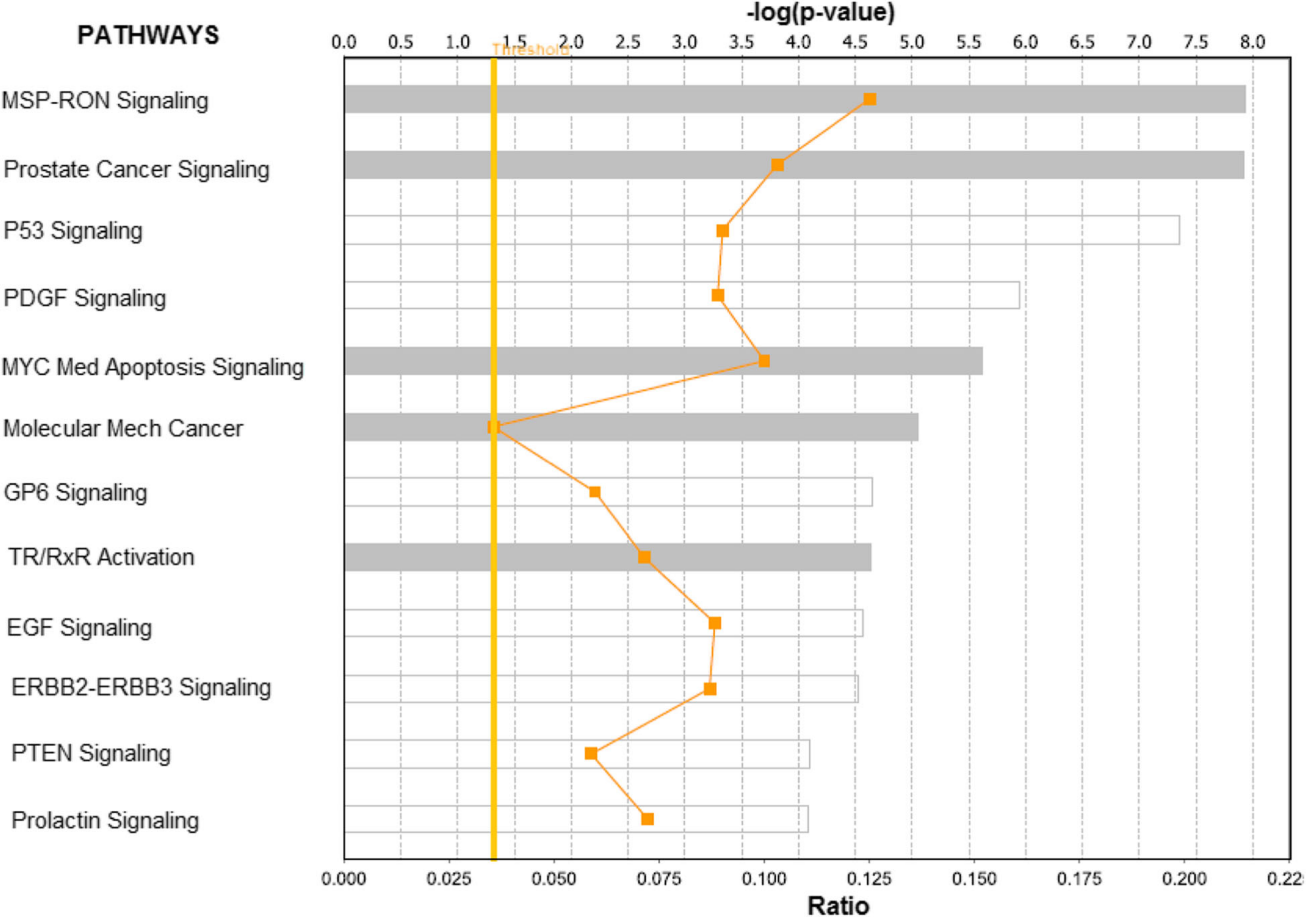
Biological Pathways enriched for germline mutations and somatic mutations mapped to genes without germline mutations. The pathways were constructed using genes containing germline mutations and highly somatically mutated genes without germline mutations. The vertical yellow line indicates the threshold level for declaring significance as determine by the log *p*-values. The zigzagging orange line indicates the ratio of molecules assigned to the pathway from input genes to the original number genes in the pathway

The results of network and pathway analysis for germline mutated genes and highly somatically mutated genes, but without germline mutations are presented in Figs. 6 and 7; respectively. Genes containing germline mutations *SERPINA3, EHF, KLK3, NCOA4, BMPR1A, RASA1, NKX3–1, TBX5, ERG, TBX3, MDM4, ATF7IP* and *CDKN2A* (Fig. 6, red fonts) were found to be functionally related and interacting with highly somatically mutated genes *TP53, EPB41L3, UTRN, AKT2, NYAP1, MYCBP2, CERK, ANK2* and *SPTBN1* (Fig. 6, blue fonts) containing no germline mutations. Pathway analysis revealed biological pathways enriched for germline and somatic mutations (Fig. 7). The most significant pathways included the MSP-RON, Prostate cancer, P53, PDGF, MYC mediated apoptosis, molecular mechanisms of cancer, GP6, TR/RxR activation, EGF, ERBB2-ERBB3, PTEN and prolactin signaling pathways (Fig. 7). Interestingly, genes containing genetic variants with strong and weak to moderate to association were found to be functionally related and interacting with highly somatically mutated genes containing no germline mutations. The functional relationships and interactions between germline mutated genes and highly somatically mutated genes with no germline mutations suggests that integrative analysis combing germline and somatic mutations information using gene expression data may partially explain the missing variation at the phenotypic level.

## Discussion

In this exploratory study, we investigated the link between germline and somatic mutation in PCa using an integrative genomics approach. The analysis revealed a signature of 124 functionally related genes containing both germline and somatic mutations. Additionally, the analysis revealed molecular networks and biological pathways enriched for germline and somatic mutations. Several studies have reported interactions between inherited polymorphism with somatic events in cancer [23] and linked germline with somatic mutations in breast cancer [24]. To our knowledge this is the first study to investigate the link between germline susceptibility variants and somatic mutations in PCa. The novel aspect of our study is that it links germline mutation information from GWAS studies with somatic mutation information from sequenced PCa tumors. Most notably, by discovering molecular networks and biological pathways enriched for germline and somatic mutations, it establishes putative functional bridges between germline-somatic mutation interactions and the biological pathways they regulate.

The clinical significance of these findings is that although PCa develops through acquired somatic driver mutations, the somatic evolution of a tumor may be significantly affected by inherited polymorphisms carried in the germline [23]. Establishing the link between germline and somatic mutations as demonstrated in this study provides a rational basis for the development of early interventions and could facilitate the realization of precision prevention in PCa. The discovery of molecular networks and biological pathways such as the androgen, P53 and PTEN signaling pathways enriched for germline and somatic mutations provides valuable insights and a framework for the development of novel targeted therapies. It is worth noting that, while we did not investigate the mechanisms by which germline and somatic mutations cooperate, the discovery of molecular networks and biological pathways enriched for the two genetic alterations tends to suggest that some form of cooperation is likely, although such cooperation could take many different forms [25]. Moreover, although we did not investigate the effects of mutations on gene expression, several studies have reported the impact of mutations on gene expression [25, 26]. Collectively, these findings emphasize the relevant of analyzing germline and somatic mutations jointly in research involving biomarker discovery in PCa.

### Limitations

Although the study provides insights about the global biological context in which germline and somatic mutations operate, limitations must be acknowledged. This study used publicly available data from genome-wide association studies and TCGA projects. GWAS has been performed almost exclusively on men of European and Asian ancestry and it is conceivable that some genetic variants may confer population-specific risks and gene and allelic expression. Studies representative of more and diverse populations are need if precision medicine and precision prevention are to be realized for the general US population. Our study did not distinguish between indolent and aggressive diseases for the reason that GWAS studies did not delineate the two clinical phenotypes. To the extent that germline and somatic alterations may differ in the two clinical phenotypes, further studies are needed to delineate the germline and somatic alterations in indolent and aggressive disease.

## Conclusions

This exploratory study established the link between germline genetic susceptibility variants and somatic alterations in PCa. The results underscore that PCa is an emergency property of molecular networks and biological pathways enriched for both germline and somatic mutations. We propose that germline mutations should be considered together with acquired somatic mutations in the discovery biomarkers in PCa. More research work is needed to understand the molecular mechanisms through which germline and somatic mutations interact and cooperate to drive tumorigenesis.

## Additional files

Additional file 1: **Table S1.** Comprehensive list of single nucleotide polymorphisms (herein called genetic variants) and genes associated with an increased risk of developing prostate cancer including published GWAS reports denoted by the PubMed ID and actual reference from which the data was extracted. (XLSX 81 kb)

Additional file 2: **Table S2.** Comprehensive list of somatic mutations, gene symbols, Ensemble gene IDs, chromosome number and type of mutation derived from the TCGA data and used in this study. (XLSX 1387 kb)

Additional file 3: **Table S3.** Comprehensive list of somatically mutated and significantly differentially expressed genes with gene symbols, *p*-value and adjusted p-value from supervised analysis and frequency distribution of somatic mutations per gene. (XLSX 367 kb)

Additional file 4: r **Table S4.** Significantly differentially expressed genes without somatic mutations. (XLSX 272 kb)

Additional file 5: **Table S5.** Comprehensive list of genes containing both germline and somatic mutation, chromosome position, SNP-ID (rs-ID), both germline and somatic mutation frequencies, GWAS and expression *p*-values and FDR. (XLSX 22 kb)

## Abbreviations

dbSNP: Single Nucleotide Polymorphism Database
FDR: False discovery rate
GDC: Genomics Data Commons
GO: Gene Ontology
GWAS: Genome-wide Association Studies
HGNC: Human Genome Gene Nomenclature Committee
ICGC: International Cancer Genome Consortium
IPA: Ingenuity Pathway Analysis software
PCa: Prostate cancer
R: R package
SNPs: Single nucleotide polymorphisms
TCGA: The Cancer Genome Atlas
TMM: Trimmed mean of M-values
